# Phytochemicals as potential inhibitors of NETosis: implications for immunothrombosis and chronic disease management

**DOI:** 10.1186/s12906-025-05233-x

**Published:** 2026-01-07

**Authors:** Chen Juanlu, Lihan Chen, Chun-Ju Sung, Shu-Chen Hsieh

**Affiliations:** 1 Institute of Food Science and Technology, National Taiwan University, No. 1, Sec. 4, Roosevelt Road, Taipei, 10617 Taiwan; 2 Institute of Fisheries Science, National Taiwan University, Taipei, Taiwan; 3 Department of Life Science, National Taiwan University, Taipei, Taiwan; 4 Health Science and Wellness Research Center, National Taiwan University, Taipei, Taiwan

**Keywords:** NETosis, Phytochemicals, Immunothrombosis, Neutrophils, Inflammation, Thrombosis

## Abstract

**Background:**

NETosis, a specialized form of neutrophil cell death, plays a dual role in immune regulation. While NET formation is essential for capturing pathogens, excessive NETosis contributes to immunothrombosis, oxidative stress, and tissue damage, affecting both acute and chronic diseases such as COVID-19, cardiovascular diseases, diabetes, cancer, and autoimmune conditions. Given the limitations of current treatments, including toxicity, high costs, and bleeding risks, phytochemicals are being explored for their therapeutic potential.

**Methods:**

NETosis gene sets were collected through published data, and followed by Gene set enrichment analysis (GSEA) to identify potential NETosis-inhibiting natural compounds from a library of 103 phytochemicals candidates. NETosis phenotype was confirmed by assessing NET formation through immunofluorescence staining and quantification. Candidate compounds were further validated in vitro using RT-qPCR to assess the expression of NETosis-related genes, including PADI4, TREM1, S100A8/A9, and CCL7. To evaluate the procoagulant consequences of NETosis, we performed a thrombin activity assay by incubating plasma with conditioned media from treated neutrophil-like cells.

**Results:**

Three phytochemicals—hesperidin, baicalin, and imperatorin—were identified as effective inhibitors of NETosis. Immunofluorescence staining confirmed NET inhibition, and RT-qPCR analysis showed significant downregulation of key genes involved in NET formation. In addition, thrombin activity was significantly reduced in plasma exposed to conditioned media from phytochemical-treated cells, indicating attenuation of NETosis-associated procoagulant activity.

**Conclusions:**

Hesperidin, baicalin, and imperatorin show promise as candidates for modulating NETosis, with implications for managing immunothrombosis and chronic diseases.

**Supplementary Information:**

The online version contains supplementary material available at 10.1186/s12906-025-05233-x.

## Introduction

NETosis is a unique form of programmed cell death in neutrophils, involving the release of DNA, histones, and antimicrobial proteins to form extracellular traps (NETs) that capture pathogens. Although NETosis was initially identified for its antimicrobial role, it is now recognized as a double-edged sword in immune regulation. While NET formation aids host defense, excessive NETosis has been implicated in immunothrombosis—a phenomenon in which immune-driven thrombotic events occur due to extensive NET formation in blood vessels. This effect has gained considerable attention in COVID-19, where severe cases often show widespread immunothrombosis and microvascular damage associated with uncontrolled NETosis [1–3].

Key steps in NETosis include PAD4-mediated histone citrullination, which facilitates chromatin decondensation [4], driven by PKCα-mediated lamin B phosphorylation [5] and CDK4/6-mediated lamin A/C phosphorylation [6]. This phosphorylation leads to lamina disassembly, a process now recognized as a key mechanistic advance in NETosis research rather than proteolytic degradation [7, 8]. Another important step is cytoskeletal disassembly, which enables extracellular chromatin expulsion [9]. Collectively, these events dismantle the nuclear and cytoplasmic architecture, allowing NET release.

Beyond acute responses, NETosis also plays a significant role in the progression of chronic diseases, including central nervous system (CNS) injuries [10], cardiovascular diseases [11, 12], diabetes [13, 14], cancer [15], long COVID [16, 17] and autoimmune diseases [18]. In these conditions, NETosis can drive oxidative stress, endothelial damage, and immune dysregulation, exacerbating disease severity and contributing to long-term complications.

For COVID-19, preventive and therapeutic anticoagulant treatments have shown efficacy in reducing mortality among hospitalized patients [19]. However, traditional anticoagulants are limited in addressing NETosis-induced immune thrombosis [20, 21], and the associated bleeding risks restrict their use in outpatient settings [22].

Despite the therapeutic potential of targeting NETs, current treatment strategies face significant challenges, including toxicity and high costs. Agents such as DNase [23], an enzyme that degrades NETs, and PAD4 inhibitors [24], which play a critical role in preventing chromatin decondensation required for NET formation, have shown promising effects. However, further clinical validation is needed to confirm their safety and efficacy [23–25].

Given these limitations, natural compounds—particularly phytochemicals—are attracting increasing attention due to their favorable safety profiles and potential as adjunct therapies. In this study, we employed gene set enrichment analysis (GSEA) to identify phytochemicals with potential NETosis-inhibiting properties. This computational approach, complemented by cellular validation assays, identified hesperidin, baicalin, and imperatorin as potential candidates, presenting new opportunities for safe and effective modulation of NETosis.

## Materials and methods

### In silico phytochemical efficacy prediction

This study began by applying Gene Set Enrichment Analysis (GSEA) to identify phytochemicals with gene expression profiles obtained from GEO Omnibus database (GSE160587, GSE85871) that could impact NETosis. The gene sets of NETosis for GSEA were derived from established scientific literature (Supplementary Table S1), in which the experimental approach focused on treating cells to induce NETosis and recording the resultant changes in protein expression through Mass Spectrometry (MS). This process led to the identification of increased gene expression following NETosis induction, which was then organized into sets as activated genes of NETosis for GSEA analysis. Using a False Discovery Rate (FDR) q-value of less than 0.25 as a benchmark for statistical significance, an extensive analysis of over 100 phytochemicals revealed several compounds with expression profiles that are opposite to the profile of NETosis, highlighting their potential as inhibitors to suppress NETosis.

### Cellular phenotype validation

#### Cell culture and DMSO-induced differentiation

The HL-60 cell line (ATCC^®^ CCL-240™), a human promyelocytic leukemia cell line, was obtained from the American Type Culture Collection (ATCC, Manassas, VA, USA). Cells were cultured in Iscove’s Modified Dulbecco’s Medium (IMDM) supplemented with 10% fetal bovine serum (FBS), 100 U/mL penicillin, and 100 µg/mL streptomycin (all from Gibco, Thermo Fisher Scientific, Waltham, MA, USA). Cells were maintained at 37 °C in a humidified atmosphere containing 5% CO_2_ and treated with 1.3% dimethyl sulfoxide (DMSO; Sigma-Aldrich, St. Louis, MO, USA) for 7 days to induce neutrophil-like differentiation.

#### PMA-induced NETs formation assay

Differentiated HL-60 (dHL-60) cells were seeded into 96-well black plates (Corning, Corning, NY, USA) at a density of 1 × 10^5^ cells per well and treated with 200 nM phorbol 12-myristate 13-acetate (PMA; Sigma-Aldrich, St. Louis, MO, USA) to induce NETosis. The control group was incubated with IMDM containing 10% FBS and an equivalent amount of DMSO as used in the treatment group. After a 4-h incubation, SYTOX Green (Thermo Fisher Scientific, Waltham, MA, USA) was added to stain cells with exposed DNA during NETosis. Fluorescence intensity was measured using a Synergy H1 microplate reader (BioTek, Winooski, VT, USA) at an excitation wavelength of 488 nm and emission wavelength of 525 nm.

#### Phytochemical treatment protocol

Hesperidin (≥ 80% purity, from *Citrus spp*.), Baicalin (≥ 90% purity, from S*cutellaria baicalensis*), and Imperatorin (≥ 95% purity, from *Angelica dahurica*) were purchased from Sigma-Aldrich, St. Louis, MO, USA. Stock solutions were prepared in DMSO and subsequently diluted with IMDM to the desired concentrations. The experimental results of Control (DMSO) and PMA treatments on dHL-60 cells are provided in the Supplementary data (Supplementary Fig. S1). dHL-60 cells were pretreated with phytochemicals (10 nM) for 1 h, followed by induction with PMA (200 nM) for an additional 4 h. NETs formation assay was performed as described in section “PMA-Induced NETs Formation Assay”.

#### Thrombin activity assay

Following the phytochemical treatment protocol, the culture medium was collected and centrifuged to remove cellular debris, retaining only the supernatant. This supernatant was then mixed with human plasma and incubated at 37 °C for 1 h. Subsequently, thrombin activity in the mixture was measured using a thrombin activity assay kit (Abcam). Briefly, 10 µL of each sample or standard was added to a 96-well plate, followed by 90 µL of assay mix containing thrombin substrate. The plate was incubated at 37 °C, and absorbance at 405 nm was measured at designated time points over 2 h. Thrombin activity was determined from a standard curve generated using serial dilutions of the thrombin standard.

#### Verification of phytochemical effects on PMA-induced NETosis in dHL-60 cells via RT-qPCR

dHL-60 cells were treated as described in Section “Phytochemical Treatment Protocol”. Following treatment, total RNA was extracted using TRIzol reagent (Invitrogen, Thermo Fisher Scientific, Waltham, MA, USA) in accordance to the manufacturer’s instructions. Complementary DNA (cDNA) was synthesized from 1 µg of total RNA using the RevertAid First Strand cDNA Synthesis Kit (Thermo Fisher Scientific, Waltham, MA, USA).

Real-time quantitative PCR (RT-qPCR) was performed using the PowerUp SYBR Green Master Mix (Applied Biosystems, Thermo Fisher Scientific) on a QuantStudio 3 Real-Time PCR System (Applied Biosystems, Thermo Fisher Scientific, Waltham, MA, USA). The PCR analysis targeted specific genes, including TREM1, S100A8, S100A9, CCL7, and PADI4 (primer sequences are provided in Supplementary Table S2). Glyceraldehyde 3-phosphate dehydrogenase (GAPDH) was used the reference gene for normalization and relative gene expression was calculated using the 2^−ΔΔCt^method [26].

### Statistical analysis

Data are presented as mean ± standard deviation (SD) from at least three independent experiments. Statistical significance was assessed using one-way analysis of variance (ANOVA) followed by Tukey’s post hoc test. p-value < 0.05 was considered statistically significant. All statistical analyses were performed using GraphPad Prism 8 (GraphPad Software, San Diego, CA, USA).

## Results

### Identification of potential phytochemicals inhibiting NETosis using GSEA

To identify phytochemicals with potential NETosis-inhibiting properties, we first performed Gene Set Enrichment Analysis (GSEA) using a gene set derived from NETosis-related proteins identified through mass spectrometry analysis (Supplementary Table S1). Next, we obtained transcriptomic expression profiles of 103 phytochemicals from the NCBI GEO Omnibus database (GSE160587, GSE85871) and standardized the data into GSEA compatible formats. Finally, phytochemicals demonstrating inverse gene correlation with the NETosis gene set were selected as candidates for further analysis. After excluding compounds with acquisition challenges, high market value, or significant toxicity, we selected three botanical constituents from medicinal materials that are affordable and accessible for traditional use, namely hesperidin, imperatorin, and baicalin.

**Fig. 1:**
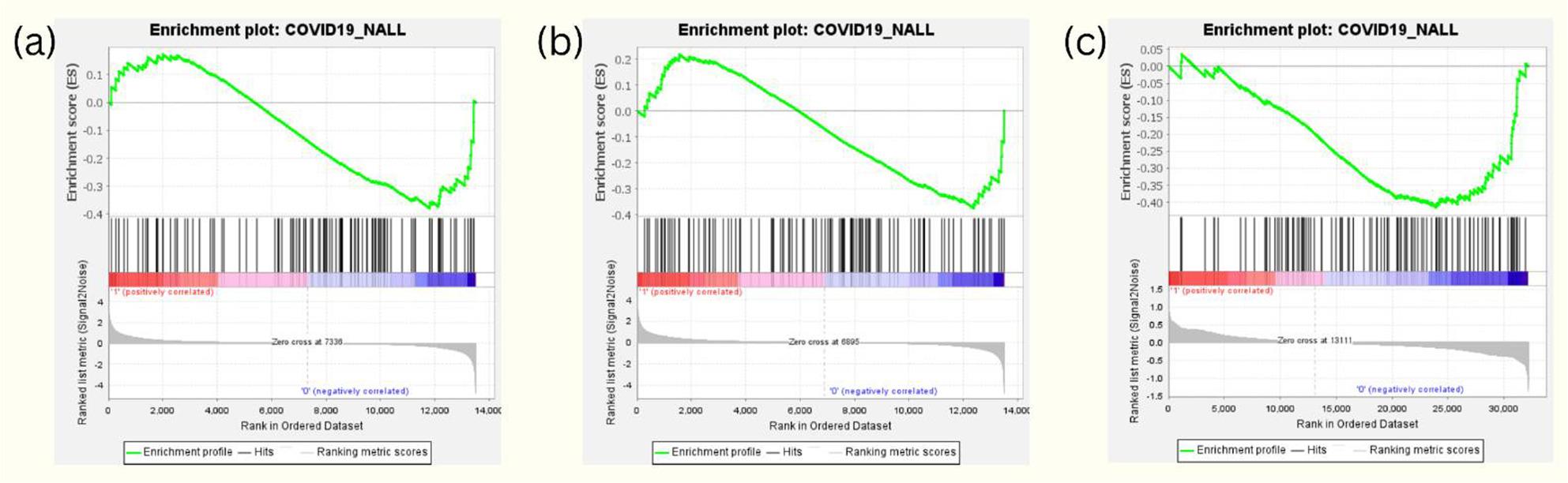
Gene Set Enrichment Analysis (GSEA) results for potential NETosis inhibitors. GSEA results show negative association between NETosis-related gene set and gene expression profiles of (**a**) hesperidin, (**b**) imperatorin, and (**c**) baicalin

GSEA results revealed that the gene expression profiles of hesperidin (Fig. 1a), imperatorin (Fig. 1b), and baicalin (Fig. 1c) exhibited a negative association with the transcriptomic expression levels of the NETosis transduction pathway, suggesting their potential role in inhibiting NETosis.

### Validation of the NETosis inhibitory effect of GSEA-identified phytochemicals in cell-based assays

To validate the NETosis-inhibiting effects of hesperidin, imperatorin, and baicalin, we employed a phorbol 12-myristate 13-acetate (PMA)-induced NETosis model in differentiated HL-60 (dHL-60) cells. NETosis was assessed by quantifying total cell number with the morphology of NETosis and measuring fluorescence-stained DNA exposed in NETs using a microplate reader.

**Fig. 2:**
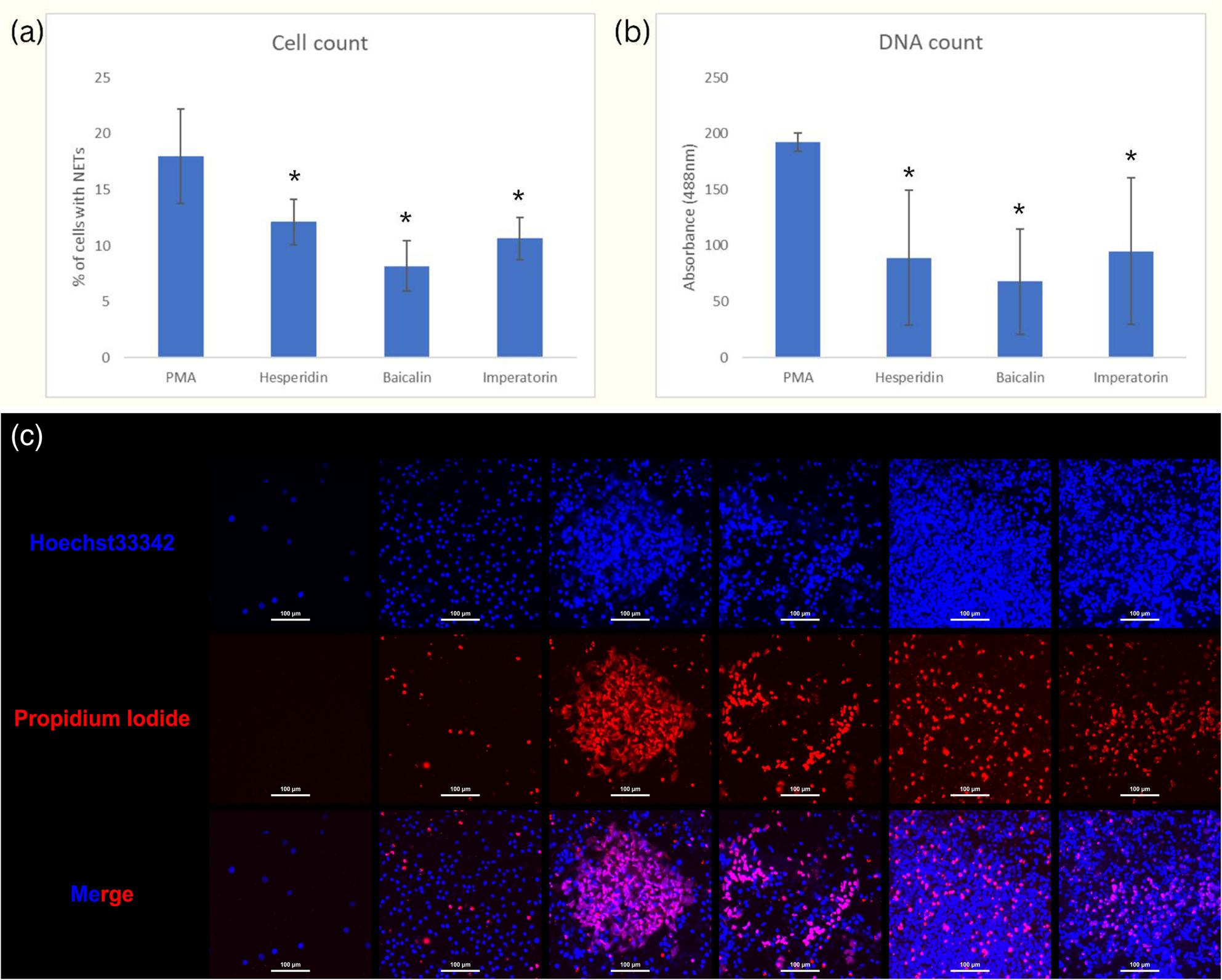
Validation of NETosis inhibition by identified phytochemicals in differentiated HL-60 cells. **a** Quantification of cells displaying NETosis-specific morphology after treatment with hesperidin, imperatorin, or baicalin compared to PMA-induced control. **b** Fluorescence intensity measurement of exposed DNA stained by SYTOX Green in NETs. Data are presented as mean ± SD from three independent experiments. * indicates *p* < 0.05, representing a significant difference compared to the PMA-induced control group. **c** Representative images of NETosis in DMSO-differentiated HL-60 cells pretreated with different phytochemicals and stimulated with PMA. HL-60 cells were differentiated using 1.3% DMSO for 5 days and then pretreated for 1 hour with one of three phytochemicals—hesperidin, baicalin, or imperatorin—before stimulation with 200 nM phorbol 12-myristate 13-acetate (PMA) for 4 hours. To assess NETosis, cells were stained with Hoechst 33342, which penetrates both live and dead cells to label nuclear DNA, and propidium iodide (PI), which only stains extracellular or membrane-compromised DNA. The appearance of extracellular PI-positive DNA indicates the occurrence of NETosis. Representative images are shown for each treatment condition

Treatment with hesperidin, imperatorin, or baicalin significantly reduced NETosis levels compared to the PMA-induced control, as evidenced by a decreased number of cells exhibiting NETosis-specific morphology (Fig. 2a) and a reduced fluorescence signal indicating extracellular DNA exposure (Fig. 2b).

In addition to quantitative assessments, we performed fluorescence microscopy to visually confirm the inhibitory effects of the selected phytochemicals on NETosis. HL-60 cells were differentiated using DMSO and then pretreated with hesperidin, baicalin, or imperatorin before PMA stimulation. Cells were stained with Hoechst 33,342 and propidium iodide (PI) to distinguish nuclear and extracellular DNA. In control cells stimulated with PMA, abundant extracellular PI-positive DNA structures characteristic of NETs were observed. In contrast, cells treated with any of the three phytochemicals showed markedly reduced PI-positive extracellular DNA, indicating inhibition of NET release. Representative images are shown in Fig. 2c, which support the quantitative findings and confirm the morphological suppression of NETosis by these compounds. 

### Effects of hesperidin, imperatorin, and baicalin on NETosis-related gene expression

To provide molecular evidence for the NETosis-inhibiting effects of hesperidin, imperatorin, and baicalin, we examined the expression of NETosis-related genes (TREM1, S100A8, S100A9, CCL7, and PADI4) in the PMA-induced NETosis cell model using RT-qPCR.

**Fig. 3:**
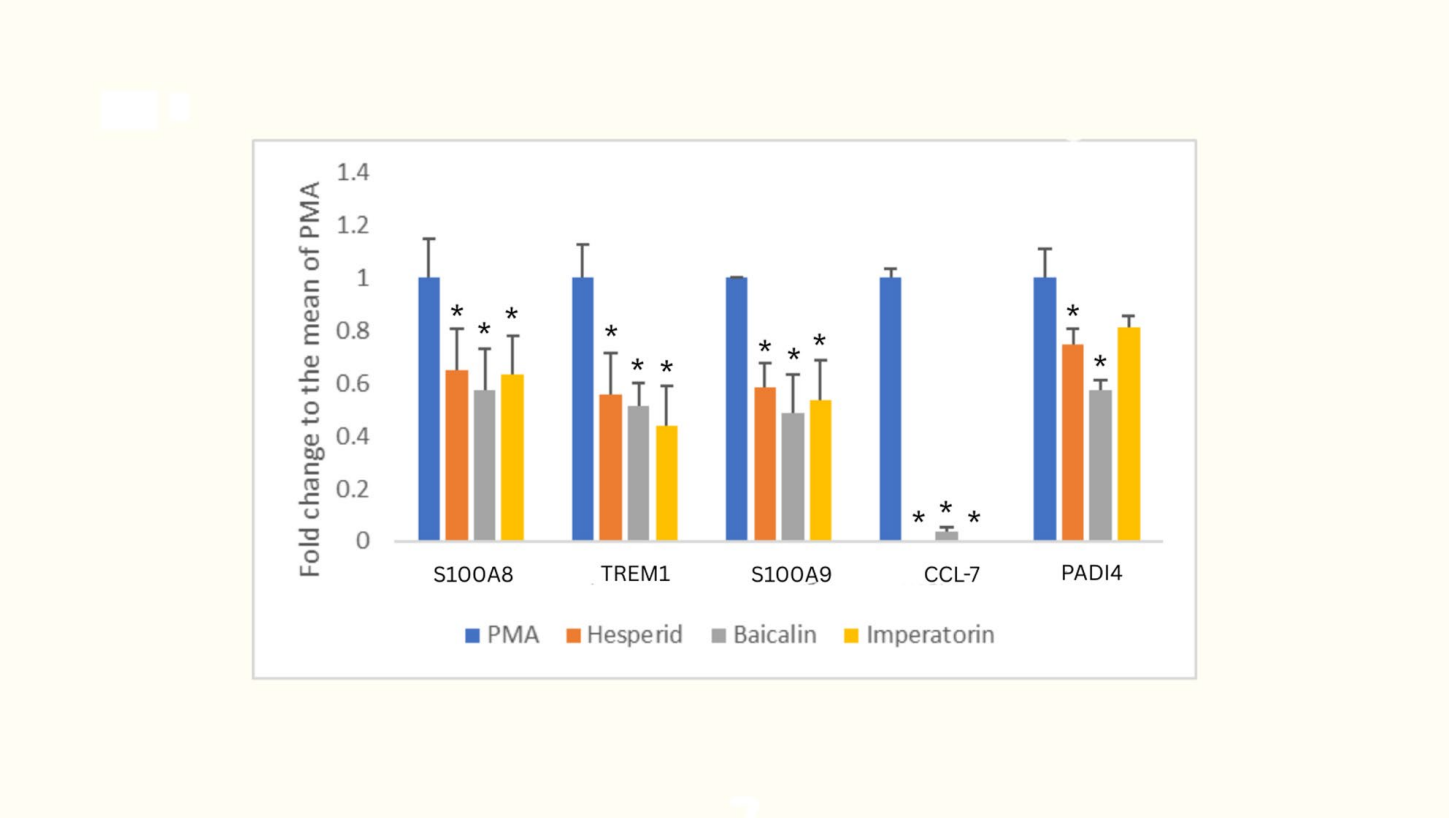
Effects of hesperidin, imperatorin, and baicalin on NETosis-related gene expression. RT-qPCR analysis of NETosis-related genes (S100A8, TREM1, S100A9, CCL7, and PADI4) expression in differentiated HL-60 cells treated with hesperidin, imperatorin, or baicalin compared to PMA-induced control. Data are normalized to GAPDH and presented as mean ± SD from three independent experiments. * indicates *p* < 0.05, signifying a significant difference from the PMA-induced control group

As shown in Fig. 3, hesperidin, imperatorin, and baicalin significantly suppressed the mRNA expression of TREM1, S100A8, S100A9, and CCL7 compared to the PMA-induced control. Notably, PADI4 expression was significantly reduced by hesperidin and baicalin, whereas its reduction by Imperatorin did not reach statistical significance.

### Inhibition of NETosis-associated thrombin activity by phytochemical treatment

To further assess the functional relevance of NETosis inhibition, we evaluated the procoagulant activity of NET-rich conditioned media by measuring thrombin activity in human plasma. Differentiated HL-60 cells were pretreated with hesperidin, baicalin, or imperatorin prior to PMA stimulation to induce NETosis. The resulting conditioned media were incubated with plasma, and thrombin activity was quantified as an indirect marker of NET-associated thrombus formation.

**Fig. 4:**
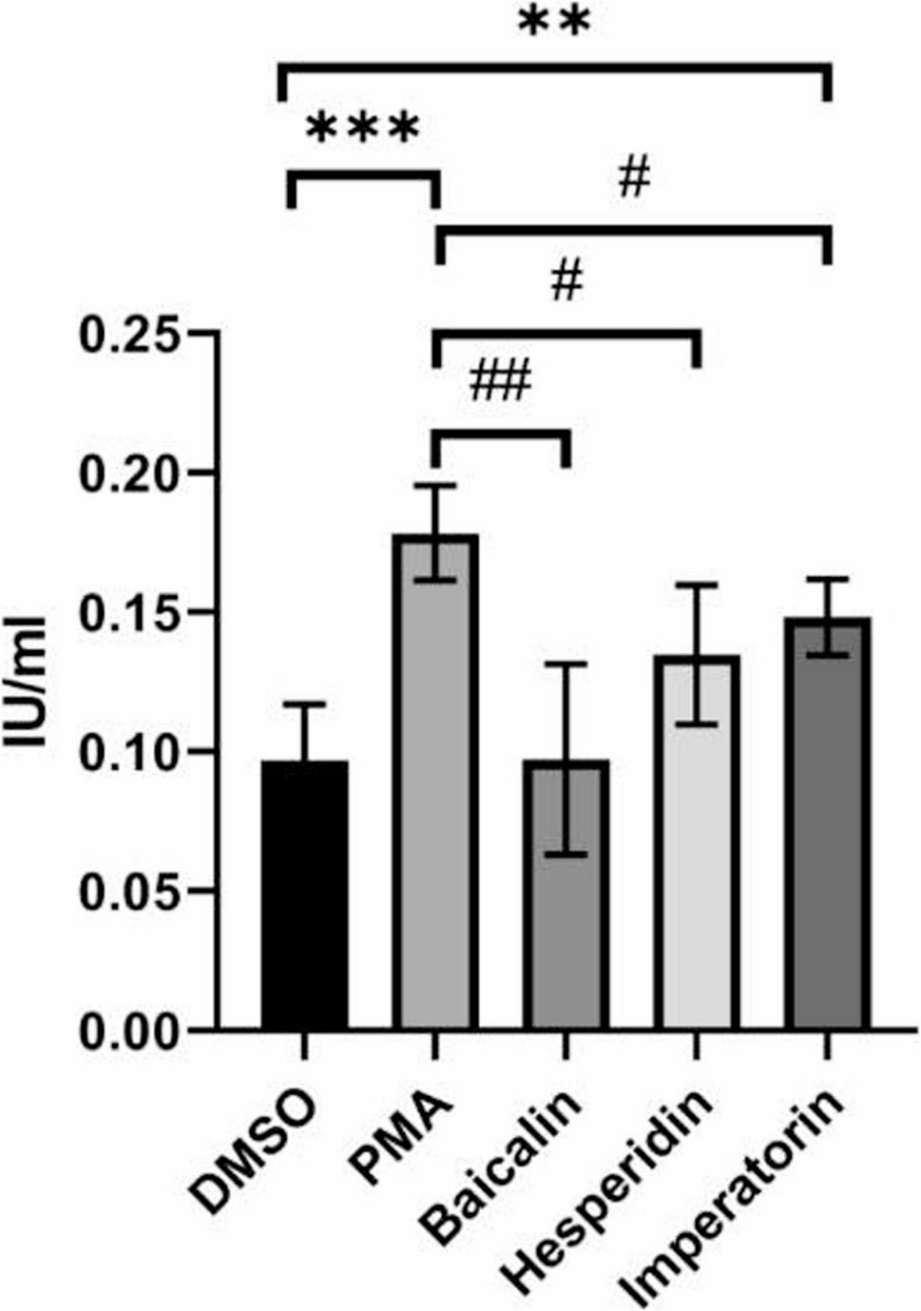
Evaluation of thrombin activity in plasma after exposure to conditioned media from differentiated HL-60 cells undergoing NETosis with or without phytochemical pretreatment. HL-60 cells were differentiated with 1.3% DMSO for 5 days and pretreated with hesperidin, baicalin, or imperatorin for 1 hour prior to stimulation with 200 nM PMA for 4 hours to induce NETosis. The conditioned media were then collected and incubated with plasma at 37 °C for 1 hour. Thrombin activity was measured using a thrombin activity assay, serving as an indirect indicator of thrombosis formation potentially promoted by NETs. A reduction in thrombin activity indicates a suppression of NETosis-induced thrombus formation. The data shown are representative results under each treatment condition

As shown in Fig. 4, plasma exposed to conditioned media from PMA-stimulated HL-60 cells exhibited significantly increased thrombin activity compared to unstimulated controls, indicating NETosis-induced prothrombotic effects. Notably, pretreatment with hesperidin, baicalin, or imperatorin led to a marked reduction in thrombin activity, suggesting that these phytochemicals not only suppress NET formation but also attenuate downstream thrombotic potential. These findings support the therapeutic potential of the compounds in mitigating NETosis-associated immunothrombosis.

## Discussion

Despite the pathogens-capturing function of NETosis, excessive NETosis is associated with various adverse outcomes, including autoimmunity, inflammation, and tissue damage [27]. Although the involvement of NETosis in both acute and chronic diseases has been established, the molecular mechanisms underlying NETosis remains largely unknown.

Using data analysis, we predicted the compounds with NETosis-inhibiting potentials from 103 phytochemicals and selected those with relatively low cost and easy accessibility for further study. Based on the results of GSEA, genes initially identified within NETosis-related gene sets and further highlighted through enrichment analysis were used for validation. Subsequent RT-qPCR analysis revealed that the three selected phytochemicals significantly downregulated genes such as TREM1, S100A8/A9, CCL7, and PADI4. Consistently, these genes have been reported to play critical roles in NET formation, underscoring their significance in NETosis regulation [28–32]. We conducted an in-depth review of the literature, revealing that PADI4, a key regulator of chromatin release and NET formation, has been linked to rheumatoid arthritis-related interstitial pneumonia [33], while TREM1 has been associated with pulmonary fibrosis [34, 35]. Additionally, CCL7, S100A8, and S100A9 have demonstrated connections to idiopathic pulmonary fibrosis [36, 37]. These associations align with the observation that NETs activation in the bloodstream can promote hypercoagulability and thrombosis, thereby exacerbating disease progression. For example, as COVID-19 transitions to an endemic stage, an increase in interstitial pneumonia cases has also been observed [38], suggesting a potential link between NETosis and pulmonary complications. Interstitial lung diseases (ILDs), such as idiopathic pulmonary fibrosis (IPF), are characterized by chronic progression and irreversible fibrosis, leading to a poor prognosis with a 3–5 year survival rate [39].

Here, we verified that hesperidin, baicalin, and imperatorin downregulated NETosis genes, including PADI4, TREM1, CCL7, S100A8, and S100A9; we also confirmed their inhibitory effects on NETs formation in a neutrophil cell model through fluorescence staining of NETs and the quantification analysis.

Differentiated HL-60 cells are widely used to study NETosis, but they do not fully mimic physiological conditions. To bridge the gap between in vitro assays and clinical relevance, we performed thrombin activity assay using human plasma. The results demonstrated that phytochemical-mediated inhibition of NETosis was closely associated with a marked reduction in procoagulant activity, thereby supporting the clinical potential of these compounds.

Our identification of phytochemicals as potential NETosis inhibitors should be interpreted in the context of these recent mechanistic advances. NET formation involves a tightly regulated cascade of intracellular events. Histone citrullination by PAD4 reduces histone-DNA affinity, promoting chromatin relaxation [1]. Nuclear envelope rupture is mediated by the phosphorylation of lamin B, driven by PKCα nuclear translocation or CDK4/6 activation, which results in lamina disassembly rather than proteolytic degradation [3]. This step allows decondensed chromatin to traverse the nuclear envelope. Additionally, cytoskeletal disassembly is required for the extracellular expulsion of DNA [4]. Our results suggest that the natural compounds tested may exert their NETosis-inhibitory effects by interfering with upstream signaling events, such as gene regulation or post-translational modifications (e.g., PAD4 or PKCα-mediated pathways). Elucidating the exact molecular interactions between these phytochemicals and NETosis-related pathways will be important for evaluating their therapeutic relevance.

There are many causes of NETosis, including pathogens, inflammation, oxidative stress and endogenous danger signals [40]. However, NETosis can further amplify oxidative stress, inflammation, and clotting, thereby exacerbating the disease conditions [41]. In CNS injuries, such as traumatic brain injury (TBI) and spinal cord injury (SCI), NETs exacerbate oxidative stress and neuroinflammation, hindering neuronal repair and recovery [42, 43]. Additionally, NETosis-induced inflammation is closely associated with complications in diabetes and cancer, underscoring the therapeutic potential of targeting NETs in these chronic conditions [13, 14, 44, 45].

It has been reported that the suppression of TREM-1 reduces NETosis and alleviates endothelial dysfunction, which may subsequently mitigate NETosis-related inflammation [29]. Similarly, the downregulation of S100A8/A9 would reduce inflammation in NETosis, particularly in aggressive cancers and thrombotic diseases [46–48]. Furthermore, CCL7, a key contributor to cytokine storms, is implicated in NETosis-related complications and may influence disease outcomes [31]. Finally, PADI4, often linked to autoimmune diseases, play a s potential role in inflammatory modulation by preventing histone citrullination essential for chromatin decondensation [28].

In this study, hesperidin, baicalin, and imperatorin suppressed TREM1, S100A8/A9, and CCL7. The documented anti-inflammatory and anti-oxidative properties of hesperidin, baicalin, and imperatorin offer added therapeutic potential [49–51]. By simultaneously inhibiting NETosis and mitigating inflammation and oxidative stress, these compounds present a promising, multi-targeted approach for managing NETosis-related diseases. This combined mechanism of action may enhance their efficacy in treating conditions associated with excessive NET formation.

To contextualize the potential relevance of the phytochemicals identified in this study, we reviewed representative NETosis inhibitors previously reported in the literature. Among the most extensively studied is Cl-amidine, a non-selective peptidylarginine deiminase (PAD) inhibitor that suppresses PAD4-mediated histone citrullination, thereby blocking chromatin decondensation and subsequent NET release [52]. Another well-known compound is diphenyleneiodonium (DPI), which acts as a NADPH oxidase inhibitor by reducing reactive oxygen species (ROS) production and thereby inhibiting ROS-dependent NETosis [53].

However, these synthetic inhibitors have notable limitations. For example, Cl-amidine, although effective, suffers from potential toxicity and limited selectivity [54]. DPI, while widely used in experimental settings, exhibits broad off-target effects and disrupts mitochondrial respiration, reducing its translational applicability [55].

In contrast, the phytochemicals identified in this study—hesperidin, baicalin, and imperatorin—are natural compounds with potentially lower toxicity and improved metabolic compatibility. Our results demonstrate that these compounds significantly downregulate NETosis-associated gene expression, including TREM1, S100A8, S100A9, CCL7, and PADI4, highlighting their promise as safer modulators of NETosis for future therapeutic development.

Future research should focus on validating the effects of hesperidin, baicalin, and imperatorin using animal models. Additional investigations are warranted to evaluate pharmacokinetics, safety profiles, and dose-response relationships. Furthermore, potential synergistic effects with conventional therapies or among these phytochemicals should be explored. These efforts will strengthen the translational pathway for developing phytochemical-based interventions targeting NETosis-associated disorders.

## Conclusion

In summary, targeting NETosis holds significant therapeutic potential for managing chronic diseases such as cancer, cardiovascular conditions, autoimmune disorders, diabetes, and central nervous system injuries. Additionally, during seasonal COVID-19 outbreaks, NETosis may be considered a key factor in exacerbating immune-mediated complications. Identifying phytochemicals capable of modulating NETosis presents a promising approach for developing safer, natural therapeutic interventions with fewer side effects than conventional treatments. Furthermore, the application of in silico prediction methods may accelerate the discovery and validation of effective therapies, providing a crucial tool for addressing complex disease mechanisms in the future.

## Supplementary Information

Supplementary Material 1.Supplementary tables and figure providing additional experimental details, including compiled NETosis-related gene set lists, primer sequences, and quantitative analyses

## Data Availability

No datasets were generated or analysed during the current study.

## Abbreviations

NETosis: Neutrophil Extracellular Trap formation
NETs: Neutrophil Extracellular Traps
GSEA: Gene Set Enrichment Analysis
RT-qPCR: Reverse Transcription Quantitative Polymerase Chain Reaction
PADI4: Peptidyl Arginine Deiminase 4
TREM1: Triggering Receptor Expressed on Myeloid cells 1
S100A8/A9: S100 Calcium Binding Protein A8/A9
CCL7: C-C motif chemokine ligand 7
CNS: Central Nervous System
COVID-19: Coronavirus Disease 2019
ILDs: Interstitial Lung Diseases
IPF: Idiopathic Pulmonary Fibrosis
TBI: Traumatic Brain Injury
SCI: Spinal Cord Injury
