# Supplementary material for: Phytochemicals as potential inhibitors of NETosis: implications for immunothrombosis and chronic disease management
Source: BMC Complement Med Ther. 2026 Jan 7;26:43. doi: 10.1186/s12906-025-05233-x (PMC12870295; doi:10.1186/s12906-025-05233-x)
Supplement: Supplementary file 1 — Supplementary Material 1.Supplementary tables and figure providing additional experimental details, including compiled NETosis-related gene set lists, primer sequences, and quantitative analyses [file 12906_2025_5233_MOESM1_ESM.docx]

Supplementary Material

Table S1. NETs associated proteins

| **Metabolic enzymes** | **Structural Proteins** | **Nucleosome-Associated Proteins** | **Antimicrobial Related Proteins** | **Chaperone/Support Proteins** | **Peroxisomal enzymes** | **Other enzymes / Not Classified** |
| --- | --- | --- | --- | --- | --- | --- |
| TALDO1 | CORO1A | LYZ | LTF | ANXA1 | PRDX2 | PRTN3 |
| TKT | ACTA2 | ANXA5 | SERPINB | ANXA3 | RETN | PLBD1 |
| GPI | ARPC1B | HIST1H4A | LCN2 | HSPA8 | CAT | CHI3L1 |
| ALDOA | PFN1 | HIST3H3 | CAMP | HSPA1A | PADI3 | MMP8 |
| TPI1 | MYL6B | ANP32A | ELANE | HSPA1L | FTH1 | PPIB |
| ENO1 | GSN | NAA38 | PSMA1 | MMP9 | SOD1 | SERPINA3 |
| PGK1 | ACTN4 | S100A4 | MPO | HSPE1 | SH3BGRL3 | QSOX1 |
| GAPDH | MSN | ARHGDIB | HNE | PPIA | GSTP1 | SERPINA1 |
| LDHB | ACTR3 | H2A | PADI3 | PRDX1 | PADI4 | CTSC |
| LDHA | CAPZA1 | H2B | UBA52 | SET | HCK | CLEC4E |
| PGAM1 | ACTB | H3 | CTSG | ANXA4 | FTL |  |
| MDH1 | ACTN1 | H4 | PGLYRP4 | ANXA6 | YWHAG |  |
| MDH2 | FLNA | MNDA | EPX | HSPA2 | YWHAE |  |
| TKTL | MYH | CLC | AZU | HSPA5 | YWHAB |  |
| PPIase | LCP1 | ECP | LYZ |  | DKFZp686B04128 |  |
| KRT-10 | HMGB2 | S100A4 | AZGP1 |  |  |  |
| RAC2 | VIM | HMGN2 | S100A12 |  |  |  |
| NCF2 | CFL-1 | HP1BP3 | BPIB2 |  |  |  |
| TMSB4X | ELA2 | CRISP | HPX |  |  |  |
| LSP1 | PR3 | DEFA-1/3 | C3 |  |  |  |
| TPM2 | IQGAP1 | VCL | S100A8 |  |  |  |
| S100A9 | A1BG | GRN |  |  |  |  |
| CAP1 | LL-37 | GMFG |  |  |  |  |
| TREM1 | BASP1 | C1QB |  |  |  |  |
| C1QC | LGALS9 | CCL7 |  |  |  |  |
| CCL8 | CEACAM1 | SIGLEC14 |  |  |  |  |
| CXCL16 |  |  |  |  |  |  |

Table S2. Primer sequences used in RT-PCR analysis

| **Gene** | **Forward Sequence (5' to 3')** | **Reverse Sequence (5' to 3')** |
| --- | --- | --- |
| S100A8 | ATGCCGTCTACAGGGATGACCT | AGAATGAGGAACTCCTGGAAGTTA |
| TREM1 | CGATGTCTCCACTCCTGACTCT | CAGCAAACAGGACAGAGAAGACC |
| S100A9 | GCACCCAGACACCCTGAACCA | TGTGTCCAGGTCCTCCATGATG |
| MCP3(CCL7) | ACAGAAGGACCACCAGTAGCCA | GGTGCTTCATAAAGTCCTGGACC |
| PADI4 | ACGCTGCCTGTGGTCTTTGACT | ACCTCCAGGTTCCCAAAGGCAT |


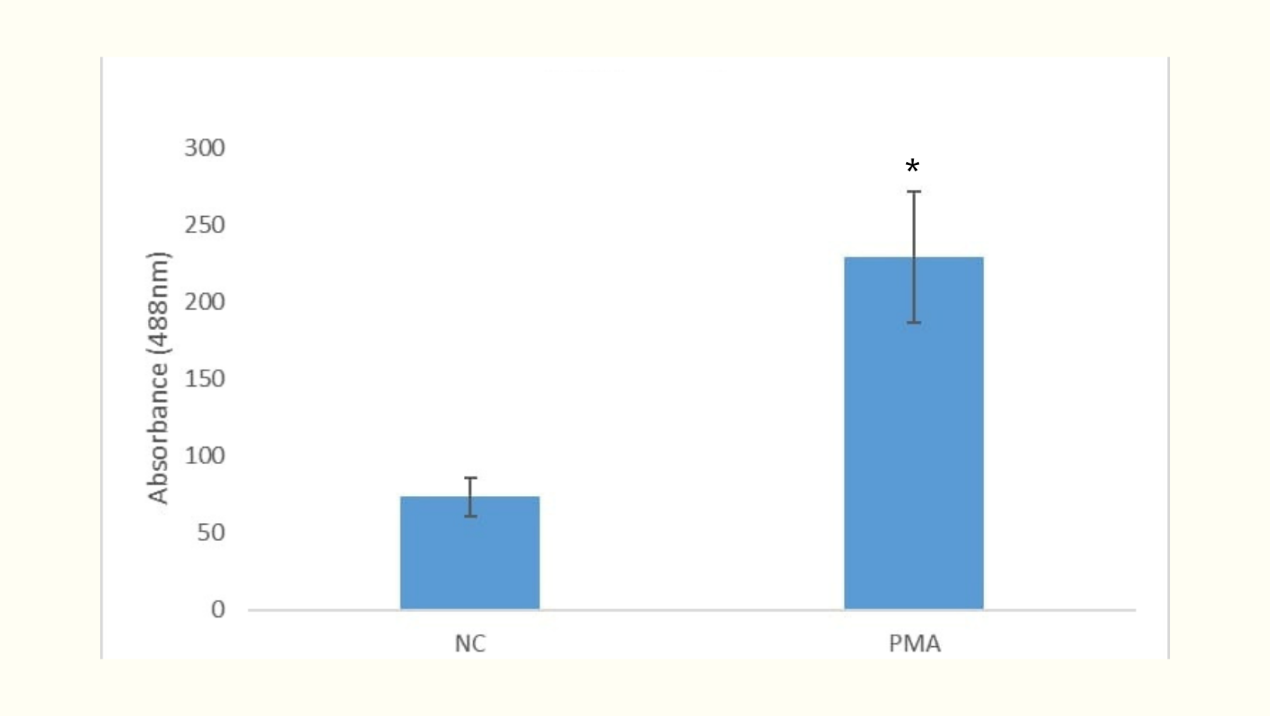


Fig. S1. DNA quantification of differentiated HL-60 cells treated with PMA to induce NETosis (positive control). dHL-60 cells were seeded into 96-well plates and treated with 200 nM PMA or an equal amount of DMSO (negative control, NC) for 4 h. Exposed DNA during NETosis was stained with SYTOX Green, and the fluorescence intensity was measured at an excitation/emission wavelength of 488 nm. Data are presented as mean ± SD (n = 3). * indicates p < 0.05 compared to the negative control.
